# Cuff Failure of Spiral-Filled Polyvinyl Chloride Endotracheal Tube Immediately after Tracheal Intubation Using a Channeled Videolaryngoscope (Pentax Airway Scope)

**DOI:** 10.1155/2020/3658092

**Published:** 2020-03-10

**Authors:** Koichi Nishikawa, Yoshihisa Fujita

**Affiliations:** ^1^Department of Disaster and Comprehensive Medicine, Fukushima Medical University, Fukushima City, Japan; ^2^Department of Anesthesiology, Northern Fukushima Medical Center, Date City, Japan; ^3^Department of Medical Engineering, Kawasaki University of Medical Welfare, Kurashiki City, Japan

## Abstract

We report on a case of mechanical damage to the spiral-filled polyvinyl chloride endotracheal tube that occurred shortly after tracheal intubation using a channeled videolaryngoscope (Pentax airway scope). We also found this problem in two other cases among 350 neurosurgery patients over the past 5 years. Prior to intubation, we did not observe any defect in the cuff. However, the cuff could not be filled with air immediately after the intubation. Anesthesiologists should be aware that, during tracheal intubation using an airway scope, friction between the endotracheal tube and inner surface of the introducer might result in sudden rupture of the cuff.

## 1. Introduction

An endotracheal tube (ETT) cuff leak due to structural damage may cause life-threatening ventilatory failure during general anesthesia [1]. We have experienced three cases of pinhole-like damage to the high-volume and low-pressure cuff of spiral-filled polyvinyl chloride (PVC) ETTs, shortly after tracheal intubation using an airway scope (AWS) (PENTAX-AWS, Hoya Corporation, Tokyo, Japan). This relatively new type of videolaryngoscope allows us indirect visualization of the vocal cords on a color monitor display and, therefore, enables tracheal intubation without the upward lifting force required to expose the glottis [2]. The bent structure of the introducer, equipped with a tube channel, provides the tube guiding function to facilitate tracheal intubation. As we previously reported [3], the AWS offers reduced hemodynamic stimulation compared with the Macintosh laryngoscope. These data suggest that tracheal intubation with the AWS is advantageous to prevent hypertension after laryngoscopy, especially in neurosurgery patients. In addition, a review of the literature [4] revealed no major complications with the AWS because of the lack of a stylet, and the continuous observation of the intubation procedure reduces the risk of oral and pharyngeal injury, supporting why we use the AWS for the tracheal intubation for neurosurgical patients. Written informed consent for publication was obtained from the patient.

## 2. Case Presentation

**Figure 1:**
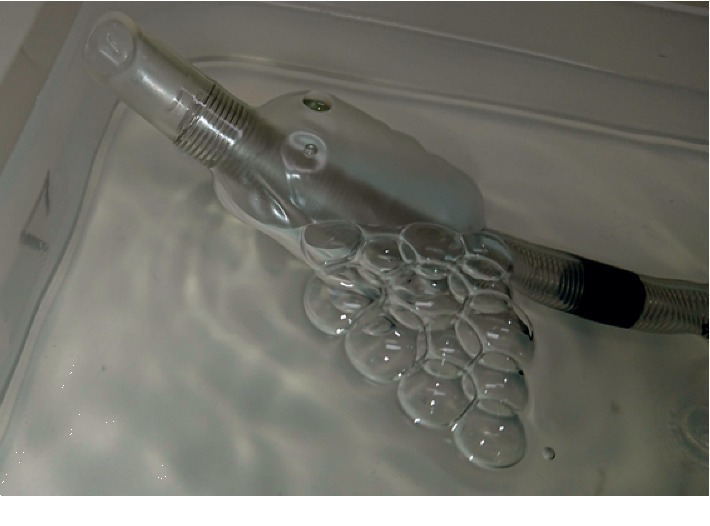
Ruptured cuff of the used ETT. When the air was inflated, air bubbles were leaking from the single, ruptured pinhole of the cuff.

A 43-year-old woman (weight 99 kg, height 176 cm) was admitted to the hospital with a headache and difficulty walking. The patient was diagnosed with a cerebrum tumor and was scheduled to undergo radical tumorectomy under general anesthesia. The patient was obese (Body Mass Index, 32.0 kg/m^2^) and had slight hypertension (155/85 mmHg, HR 92 bpm) preoperatively. There were no abnormal findings in the oral cavity, respiratory tract, and chest radiograph. After preoxygenation with 100% oxygen, general anesthesia was induced with remifentanil (0.1 *μ*g/kg/min), fentanyl (0.1 mg), propofol (150 mg), and rocuronium bromide (90 mg); the patient then underwent endotracheal intubation with a spiral-filled PVC ETT (internal diameter 7.5 mm, external diameter 10.2 mm, RUSCH murphy spiral tracheal tube, 104202-75SR, Teleflex Medical Sdn. Bhd., Malaysia) using the AWS-S100. A single-use introducer (NK PBLADE ITL-SL, a standard model for use on adults), which generally accepts an ETT external diameter of 8.5–11.0 mm, was used for tracheal intubation. The patient was able to fully open his mouth (Mallampati Grade I). It is generally difficult to insert the introducer into the oral cavity of obese patients due to bulky design of Pentax blade, especially when lifting laryngoscopy for elevation of epiglottis and obtaining laryngeal view, but this procedure was performed safely without damaging the teeth. The vocal cord was clearly observed on the AWS monitor, and the tube was inserted smoothly into the trachea and was fixed at the 23 cm mark. Prior to these procedures, we checked for manufacturing defects on the ETT, but did not observe any structural problem with the cuff. Despite this, a cuff leak was suspected due to failure to sufficiently inflate the cuff and a collapsed pilot balloon; in addition, a bubbling noise showed a leak from the trachea on ventilation and indicated serious damage to the cuff system. The cuff had likely broken immediately after intubation using the AWS, as no abnormality was detected on initial inspection. We were immediately forced to replace this with a new ETT; fortunately, the reintubation was completed safe and successful using the same AWS. The surgery was performed in the prone position, and the anesthesia and surgery times were 321 min and 394 min, respectively. The postoperative pathological diagnosis was hemangioblastoma. After the operation, there was no sore throat, swelling, or edema of the upper airway. Postoperatively, the ETT was reexamined. To identify the exact sites of leakage, we attempted to inject air into the cuff with the tracheal tube immersed in water and observed air bubbles from the cuff. Only a single, very small but clearly visible pinhole-like rupture was found at approximately 10 mm distal from the proximal edge of the tracheal cuff (Figure 1). The spiral tube itself, murphy hole, inflation bulb, pilot balloon, inflating tube, and 15 mm connector were all intact.

Before this case, we experienced two other cases of similar mechanical pinhole damage to the cuff in female patients after tracheal intubation using the AWS. In these two cases, spiral-filled PVC ETTs (internal diameter 7.0 mm, external diameter 9.6 mm, RUSCH murphy spiral tracheal tube, 104202-70SR, Teleflex Medical Sdn. Bhd., Malaysia) were also used. A pinhole breakage was also observed near the proximal edge of the cuff in both cases. Reintubation was required immediately. Overall, we have experienced three cases of very similar cuff damage among approximately 350 neurosurgical patients in the past five years. In all patients, tracheal intubation was performed using the AWS.

## 3. Discussion

Tracheal intubation using the AWS-S100 was performed on approximately 350 neurosurgical patients. Among them, a serious “so-called pinhole-like rupture” of the cuff was found in three cases. Although the incidence is less than 1% (3/350), this cuff breakage can cause a life-threatening emergency and cannot be ignored. In this discussion, we will provide some guidance on how to minimize the accidental damage.

Although the cause of the structural damage of the cuff is unknown, the following factors should be considered. First, the cuff may have broken while sliding the ETT within the introducer of the AWS. Moderate or severe friction may occur between the inner surface of the introducer and outer surface of the ETT, even when sufficient lubricant has been used, because of the 90-degree bend and small internal diameter of the introducer. Cuff failure was also noted in two female patients who were intubated with smaller ETTs (internal diameter 7.0 mm and external diameter 9.6 mm). As the friction between the ETT and the introducer is the most plausible reason for the damage, it would be appropriate to check the equipment by sliding an ETT through the introducer prior to inserting it into the patient. It is interesting that the damage occurred in a similar spot (near the proximal edge) of the cuff in all three cases. Frictional pressure may be applied to this part of the cuff during tracheal intubation using the AWS. The other possibility is that the cuff was not completely deflated after the initial inspection, which could have contributed to the damage by increasing the cuff pressure during insertion. We need to deflate the cuff completely after the inspection to avoid the possibility of rupture. Furthermore, there may be very small scratches or protrusions on the inner surface of the introducer that could damage the cuff. However, it is unlikely that these scratches or protrusions, which we did not find during the preoperative inspection, broke the cuff in all three cases. Still, the inner surface of the AWS should be checked during the initial inspection.

Videolaryngoscopes vary with regard to the shape of their blades, camera location, video screen, integration of the channel for tracheal tube guidance, and single-use vs. multiple-use design. A multicenter, prospective randomized controlled trial evaluated six videolaryngoscopes (three unchanneled (C-MAC™ D-blade, GlideScope™, and McGrath™) and three channeled (Airtraq™, A.P. Advance™ difficult airway blade, and KingVision™)) in 720 patients with a simulated difficult airway [5]. There were no statistically significant differences between the videolaryngoscopes regarding side effects such as hoarseness, sore throat, dysphagia, or postoperative nausea and vomiting, but two cuff leaks occurred after intubation using a videolaryngoscope with a guiding channel (one Airtraq™ and one KingVision™). As the Pentax AWS-S100 is a guided videolaryngoscope, which is structurally similar to those mentioned above, this type of videolaryngoscope may cause the cuff failure by the similar mechanism. Other complications associated with the AWS are not well understood. Two cases of epiglottis malposition were reported during intubation using this device [6]. These case reports suggest that excessive pressure on the epiglottis by the introducer can infrequently happen, when the correct maneuver is used. A systematic review and meta-analysis that compared the efficacy of the AWS with that of the Macintosh laryngoscope revealed that there was no difference in the rate of oral or pharyngeal injuries during tracheal intubation [7].

It has been reported that lignocaine-base aerosol can cause blistering, pinholes, and sudden rupture of PVC cuffs [8, 9]. As a reason, the aerosol contains a swelling agent that softens the cuff and this may lead to blistering and rupture. Therefore, water-soluble gel (not aerosol) should be used in both tracheal tube and the inner surface of the blade because gel lubrication of ETT cuffs effectively minimizes the friction. Factors known to result in cuff leaks, such as cuff underinflation, cephalad migration of the ETT, misplaced orogastric or nasogastric tubes, wide discrepancy between ETT size and tracheal diameters, and increased peak airway pressure [1], were all absent.

In conclusion, anesthesiologists should be aware that, in cases of tracheal intubation using the AWS, friction between the ETT and inner surface of the introducer of the AWS might result in sudden rupture of the cuff.
